# Harnessing Non-Thermal External Stimuli for Polymer Recycling

**DOI:** 10.1021/acs.macromol.4c02690

**Published:** 2025-02-18

**Authors:** Glen R. Jones, Richard Whitfield, Hyun Suk Wang, Nethmi De Alwis Watuthanthrige, Maria-Nefeli Antonopoulou, Victoria Lohmann, Athina Anastasaki

**Affiliations:** Laboratory for Polymeric Materials, Department of Materials, ETH Zürich, Vladimir-Prelog-Weg 5, 8093 Zürich, Switzerland

## Abstract

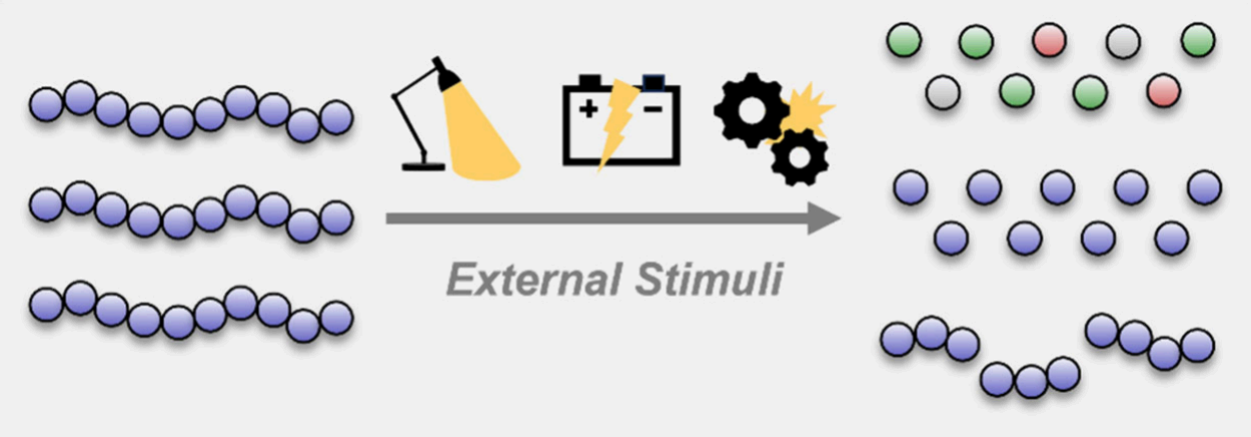

Polymeric materials have become indispensable due to their versatility and low cost, yet their environmental impact presents a significant global challenge. Traditional chemical recycling methods typically rely on heat as a stimulus; for instance, pyrolysis is a popular chemical recycling methodology which faces limitations due to high energy consumption, low product selectivity, and the generation of undesirable byproducts. In response, recent advances in the promotion of depolymerization and degradation through alternative stimuli such as light, electrochemistry, and mechanical force, have shown promising potential for more efficient and selective polymer breakdown, yielding either the starting monomers or valuable small molecules. This perspective explores key examples of these emerging strategies, highlighting their potential to improve upon current protocols and offer alternative pathways under milder conditions, while identifying significant challenges that future research must address to translate promising chemistry into viable and broadly applicable recycling strategies.

## 1 Introduction

As polymeric materials have become ubiquitous in everyday life due to their affordability, adaptability, and unique properties, plastic waste has emerged as a global problem with long-lasting repercussions that are still being uncovered. It is estimated that of the 6300 Mt of plastic waste generated up to 2015, only 9% was recycled, while the rest was either incinerated (12%) or accumulated in landfills and the natural environment (79%).^1^ Improving the sustainability of polymeric materials is therefore one of the central challenges of polymer science in the 21st century. Given the diverse nature of plastics, from their chemistry to their properties, no single solution will address this global issue. Instead, advances in mechanical recycling, chemical recycling, and the development of polymers from renewable resources are likely to form a multifaceted strategy in combination with increased governmental regulation to move the world toward a circular plastics economy.^2^ Chemical recycling in particular holds significant promise as it allows for regeneration of monomeric starting materials (as in the case of depolymerization) or generation of small molecules or oligomers (as in degradation) which could be utilized in other applications.^3^ However, because most plastics were originally designed to be resilient and durable at the lowest possible cost,^4^ breaking their robust polymer chains to obtain useful and reusable small molecules remains a significant chemical challenge, underscoring the need for innovative approaches that can selectively and efficiently trigger polymer degradation/depolymerization upon demand.

Pyrolysis (from Greek origin: pyro, “fire”, and lysis, “separating”) is the traditional and most widely used method for degrading and, in some cases, depolymerizing polymers.^5,6^ This process typically involves heating polymers to temperatures exceeding 400 °C in an oxygen-free environment, triggering thermal degradation and the generation of small molecules. Pyrolysis operates through a radical mechanism: the homolysis of the weakest bonds in the polymer’s structure initiates the breakdown.^7^ This can occur on the backbone or from scission of side groups, generating radical species.^8^ These radicals can then undergo various reactions, such as rearrangement, elimination, hydrogen abstraction, depolymerization, or termination. Ultimately, this gradual breakdown leads to progressively smaller polymer fragments, with low molecular weight compounds, often gases or liquids, being formed as the final products.^9^

Despite its simplicity, as pyrolysis does not require additional chemicals or external stimuli, it suffers from several critical drawbacks. These include high energy consumption, poor selectivity for desired products, and the formation of undesirable byproducts like solid chars and carbon dioxide. Lowering the temperature to suppress side reactions and thus increase the specificity of these processes is crucial for obtaining purer targeted molecules.^10−12^ Simply developing less thermally stable polymers is not necessarily a viable alternative for many applications, as polymeric materials are typically processed at reasonably high temperatures (∼200 °C) and additional safety considerations are necessary, for example, in the event of fire, toxic gases generated from such plastics could cause adverse health effects.^13^ A promising alternative approach is to develop new chemistries which can selectively depolymerize/degrade current commercial materials only when a specific stimulus and/or chemical conditions have been applied.

The promotion and regulation of reactions by external stimuli such as light, electrochemical, and mechanical force offers precise control over chemical transformations in a targeted and energy-efficient manner. For example, light can drive photochemical reactions which enable selective bond breaking and formation while preserving sensitive functional groups under mild conditions.^14^ Electrochemistry provides tunable control over redox processes, allowing for selective activation/deactivation of bonds with high precision.^15^ Mechanochemistry can induce bond cleavage through shear or compressive forces, facilitating reactions that may be otherwise difficult or impossible.^16^ External regulation of reactions has been utilized to great effect in polymer synthesis, where gating the activation or deactivation of monomer addition has enabled precision synthesis of complex macromolecules^17^ and spatiotemporal control.^18^ In the field of ’self-immolative’ polymers, external stimuli play a critical role in initiating depolymerization.^19,20^ These polymers are kinetically trapped in stable states until a specific trigger causes them to undergo ’unzipping’ depolymerization, efficiently breaking down into their monomeric components.^21^ This strategy allows for materials which are stable during use but can degrade rapidly and selectively when stimulated, making them useful in applications such as sensors, biomaterials, and adhesives.^22^

This perspective explores the rapidly growing field of external regulation of depolymerization and polymer degradation, with a particular focus on how these techniques are being (or could be) applied to polymeric materials currently in widespread use. Given the diverse chemistry of plastic waste, it is clear that no single approach will solve the problem; instead, a range of tailored strategies will be necessary. The diverse chemistry enabled by external stimuli such as light, electrochemistry, and mechanical force is already showing great promise in addressing this complexity. We examine how these stimuli are beginning to demonstrate remarkable selectivity under mild conditions, providing more efficient, targeted, and energy-saving pathways for degrading and depolymerizing polymers. Additionally, we discuss future areas of research that hold promise for advancing these technologies, including the development of novel chemistries and underexplored reaction mechanisms. By identifying these emerging opportunities, we aim to uncover how external regulation could drive innovations in chemical recycling, offer new solutions for transforming hard-to-degrade materials into valuable products, and contribute to a sustainable polymer economy.

For the purpose of this perspective, it is important to define key terms related to the breakdown of polymers. Depolymerization refers to the breakdown of polymers into their constituent monomers, enabling the regeneration of starting materials for new polymer synthesis. In contrast, degradation is a more random process that reduces polymers into oligomers or a range of small molecule products, often without the selective recovery of monomers. Upcycling describes the transformation of polymers into other useful materials, such as valuable small molecules or higher-value polymeric materials. These definitions provide a framework for discussing the potential of externally regulated strategies to address polymer waste.

## 2 Photoregulation

**Figure 1:**
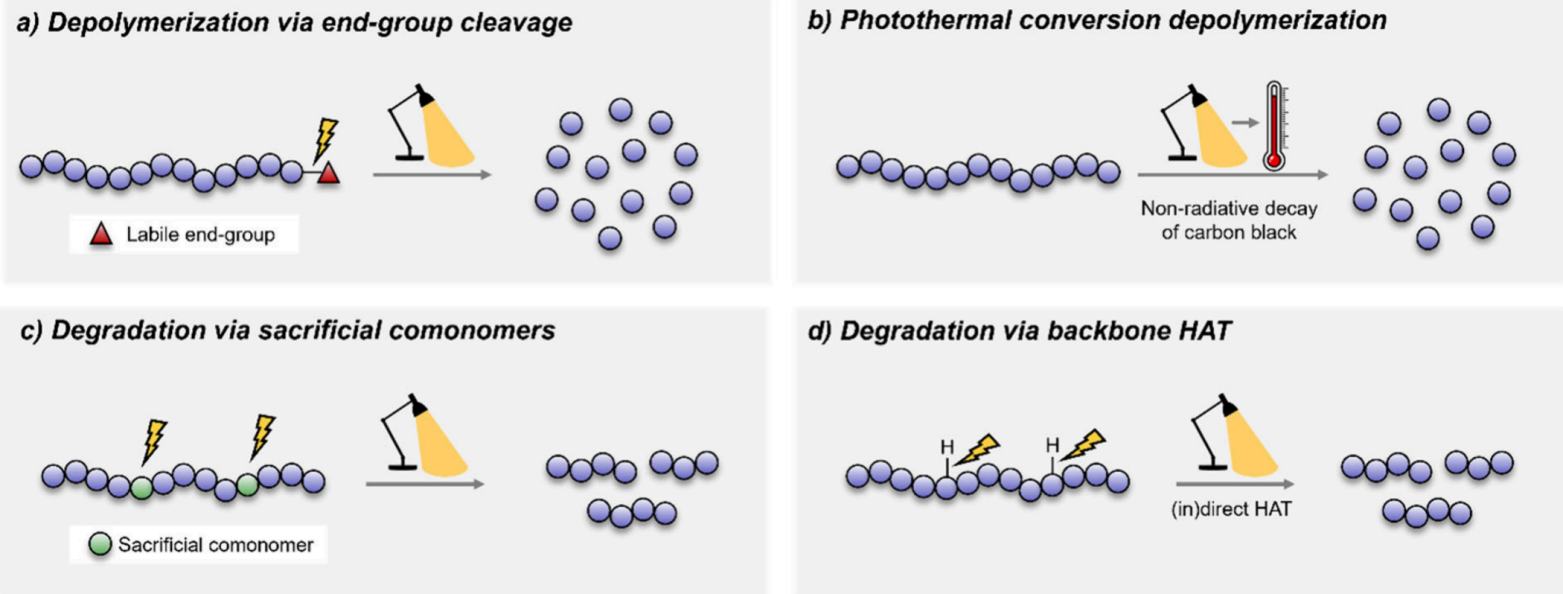
Overview of common approaches to photoregulation of depolymerization and polymer degradation.

With the exception of heat, light is easily the most widely utilized external stimulus for polymer deconstruction, if not all chemical reactions. Photoexcitation of a molecule to a thermally inaccessible high-energy state enables unique organic transformations under reduced temperatures. In the deconstruction of polymers, photoirradiation typically does not induce direct photolysis of a polymer backbone unless wavelengths <300 nm are employed. Rather, photoirradiation can indirectly trigger depolymerization/chain scission via various pathways described below (Figure 1).

**Scheme 1:**
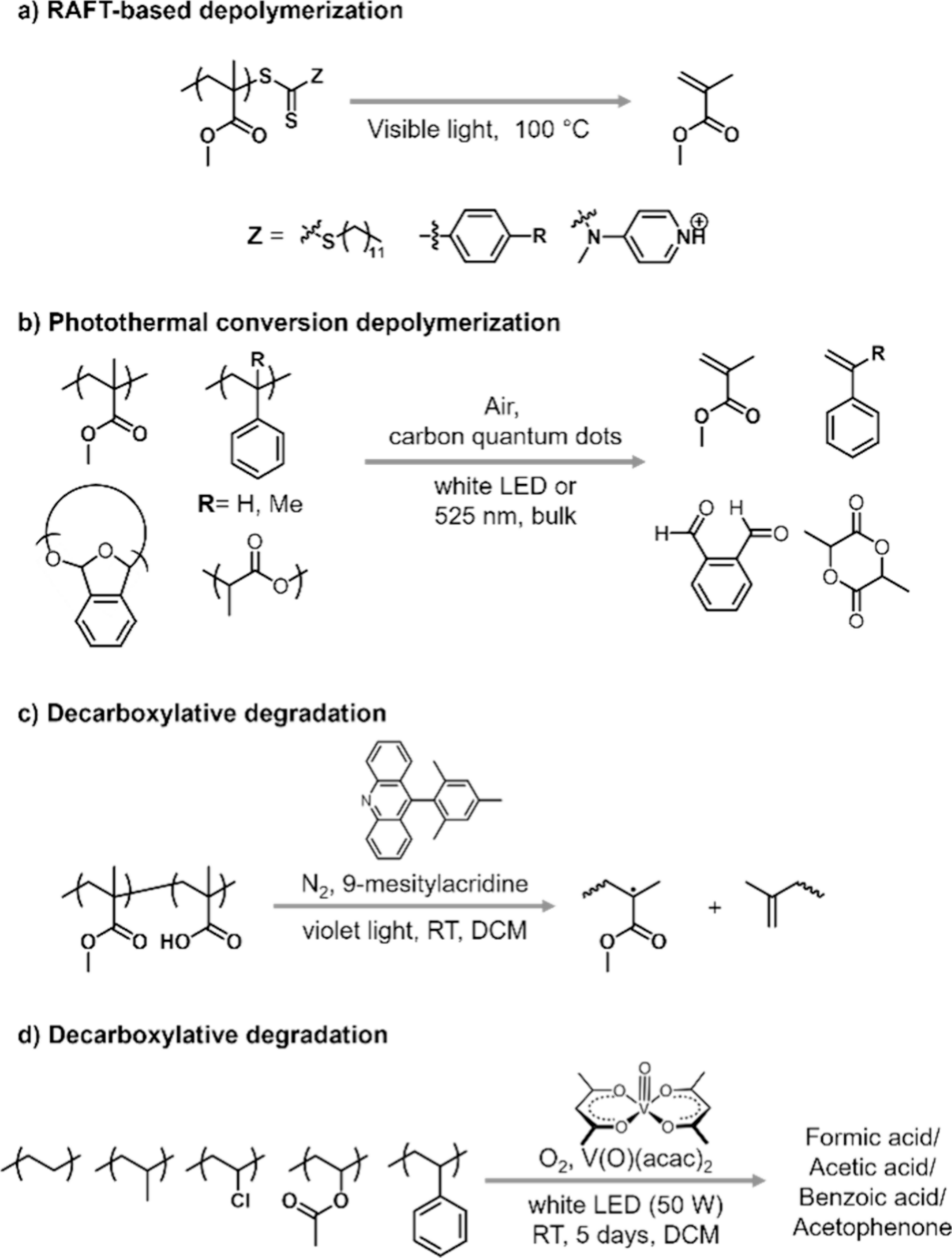
(a) Depolymerization of RAFT-Synthesized Polymethacrylates via Chain-End Activation; (b) Photothermal Conversion for Degradation/Depolymerization of a Range of Polymers; (c) Degradation of PMMA-co-PMAA via Decarboxylation; (d) Decarboxylative Degradation Using a Vanadium Catalyst and White LEDs

**Scheme 2:**
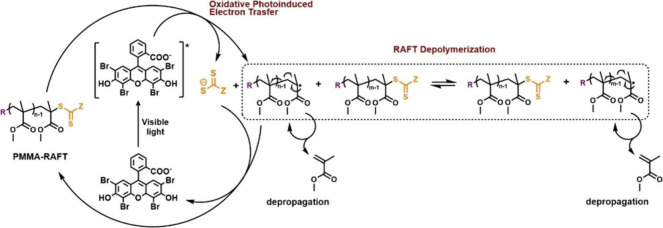
Graphical Scheme of Temporal Controlled PET-RAFT Depolymerization Demonstrating Continuous Deactivation of the Polymer Chains (Figure Partially Reproduced with Permission from Ref (27). Copyright 2023 Wiley-VCH)

The concept of intrinsically circular polymers (iCPs) was posited by Chen and co-workers in 2021, referring to chemically recyclable polymers in their kinetically trapped states.^4^ Overcoming this kinetic barrier (e.g., by reactivating a functional end-group, Figure 1a) can trigger depolymerization, provided that the reaction conditions thermodynamically favor this process (e.g., employing dilution in solvent).^23,24^ Photoirradiation has been employed by the groups of Sumerlin,^25^ Anastasaki,^26−28^ Matyjaszewski,^28,29^ and Boyer^30^ to trigger depolymerization of polymethacrylates synthesized by reversible deactivation radical polymerization (RDRP). In the most conceptually straightforward system, Sumerlin and co-workers utilized 365–515 nm irradiation to generate a chain-end radical via direct C–S homolysis of the thiocarbonylthio end-group, accelerating depolymerization to monomer under dilution and moderate (100 °C) temperatures (Scheme 1a).^25^ Independently, Anastasaki and co-workers utilized a photoinduced electron/energy transfer approach to cleave the terminal C–S bond using Eosin Y as a photocatalyst which enabled accelerated depolymerization at 100 °C even under red light irradiation (Scheme 2).^26^ In both reports, accelerated kinetics and higher final conversions were achieved when compared to purely thermal conditions in which chain-end radicals were generated presumably through solvent radicals.^31^ Delicate fine-tuning of reaction conditions enabled temporal control (i.e., “on/off” cycles) of the depolymerization.^27^ The groups of Anastasaki and Matyjaszewski also utilized photoreduction of Fe(III) to Fe(II), the latter of which could abstract the terminal Cl of polymethacrylates synthesized by atom transfer radical polymerization, generating a chain-end radical and subsequent depolymerization at 100–170 °C.^28^ It is important to note that while these techniques achieve chain activation using light, depropagation remains thermodynamically unfavorable under ambient conditions. As a result, elevated temperatures are required to make the depolymerization process thermodynamically favorable and enable significant monomer recovery. Readers interested in the thermodynamics and kinetics of vinyl polymer depolymerization are referred to a recent review on the topic.^23^

Another approach to light-triggered depolymerization by Stache and co-workers employs photothermal conversion (i.e., light to heat) agents such as carbon quantum dots to generate local hotspots that reach pyrolytic temperatures (Figure 1b).^32^ Such materials undergo largely nonradiative decay and can reach extreme surface temperatures while maintaining low bulk temperature, thereby creating sharp temperature gradients that reduce side reactions. The group investigated the depolymerization of various classes of polymers ranging from polyphthalaldehydes, polymethacrylates, poly(α-methylstyrene) (PMS), polystyrene (PS), and poly(lactic acid) (Scheme 1b).^32,33^ Remarkably, a 43% yield of styrene was achieved in the presence of BaO, demonstrating the potential of photothermal conversion in tackling plastic waste.^34^ Recent work leveraging the photothermal properties of carbon black dyes in polystyrene gave up to 60% styrene under visible light irradiation.^33^

Polymers can also be recycled by degrading the chains to more processable oligomeric fragments or converting them into small molecules that are derivatives of the original monomers. Photodegradation of acrylates and methacrylates via incorporation of a photoresponsive comonomer (Figure 1c) was demonstrated by the groups of Sumerlin,^35−37^ Seidel,^36,37^ Yagci,^38,39^ Kiliclar,^38,39^ and Ouchi.^40,41^ Sumerlin reported the photodegradation of poly(methyl methacrylate) (PMMA) containing an (*N*-acyloxyl)phthalimide comonomer using 515 nm light irradiation and Eosin Y as a photocatalyst.^35^ The comonomer could undergo N–O scission, followed by decarboxylation and backbone scission of the copolymer upon reception of an electron from a single-electron transfer (SET) donor. A similar decarboxylative degradation approach was used by the groups of Sumerlin and Seidel using acridine-based photocatalysts and methacrylic acid comonomers (Scheme 1c). This is particularly noteworthy as it allows for effective degradation of polyacrylates, a polymer class which had traditionally been difficult to break down.^36,37^ Ouchi and Nishikawa utilized a dual-stimuli approach using a base and organic photocatalyst to degrade polyacrylates containing vinyl boronate comonomers.^41^

Photocatalyzed hydrogen atom transfer (HAT) is also a promising route to polymer degradation and upcycling (Figure 1d).^42−44^ For the conversion of PS to value-added molecules, the most utilized strategy involves HAT (either direct or indirect) of the activated benzylic hydrogen on the polymer backbone in the presence of oxygen.^34,45,46^ A photocatalytically generated HAT agent (e.g., excited photocatalyst or reactive radical) introduces a backbone radical that triggers β-scission of the backbone C–C bond via a peroxy radical intermediate, resulting in oxygen-containing aromatics such as benzoic acid. Das and co-workers employed *N*-bromosuccinimide and sodium trifluoromethanesulfinate to produce up to 73% of benzoic acid from PS, representing the highest product yield to date.^47^ Photocatalyzed HAT is also promising for both recycling and functionalization of polyolefins and other vinyl polymers containing C(*sp*^3^)–H backbone bonds. Soo and co-workers employed a vanadium-catalyzed (V(O)(acetylacetonate)_2_) tandem C–H oxidation and C–C scission to produce various high-value molecules from polyolefins, PS, poly(vinyl chloride), poly(vinyl acetate) (PVAc), and poly(ethylene-vinyl acetate) (Scheme 1d).^48^

Clearly, employing light (particularly sunlight) as an external stimulus for polymer deconstruction is highly promising. Some of the main challenges for implementation at the industrial scale include scale-up without compromising light penetration, particularly for reactions that proceed in the absence of solvent. Another key challenge is to improve product selectivity for processes that employ HAT randomly across the polymer backbone. By developing HAT reagents that can target specific protons on a polymer backbone, desired reactions could be promoted.^49^ Utilization of the oligomeric fragments resulting photodegradation is also still in the early stages and must be addressed to maximize the economics of such reactions. Finally, and perhaps most importantly, lifecycle analyses (LCAs)^50^ for these light-driven processes have not yet been conducted. Even if more efficient chemistries are developed, it is essential that the LCA demonstrates a favorable environmental and economic outcome compared to existing pyrolytic methods for these approaches to be considered viable alternatives.

## 3 Electrochemical Regulation

Modern electrochemical synthesis has enabled many advances in the synthesis of small molecules over the past 20 years,^15^ and has also been exploited for exerting control over polymer synthesis.^51^ The specific advantage of electrochemistry is the precise control over the potential energy of electrons afforded using applied voltage, allowing for reactions that other techniques cannot achieve, often under milder conditions.^52^ Compared to other external stimuli discussed in this perspective, the utilization of electrochemistry to selectively degrade and depolymerize polymers is relatively less explored, although it has experienced rapid progress in the past few years.^53,54^ This section highlights key examples of these approaches and explores potential future applications of electrochemistry.

**Figure 2:**
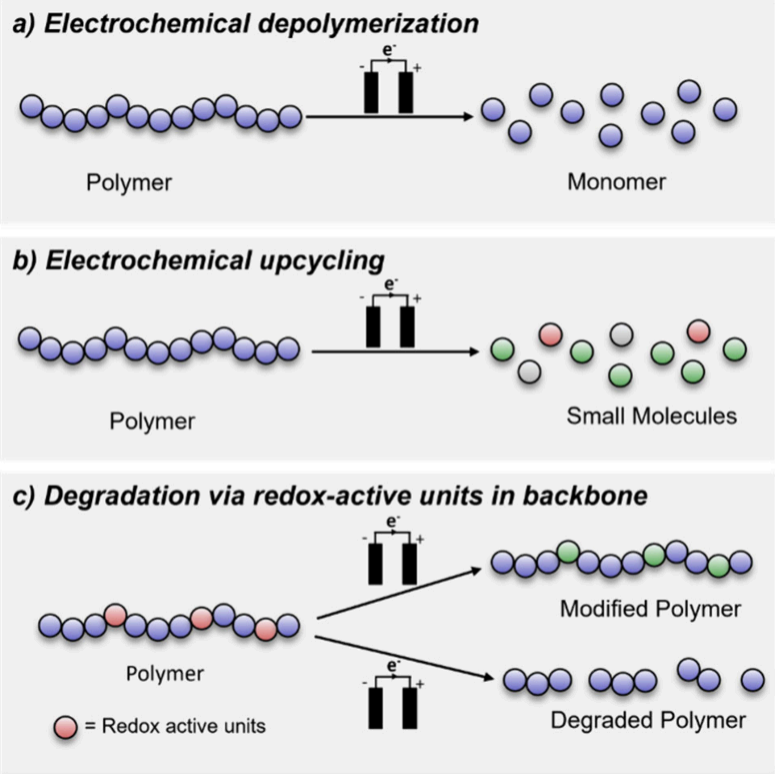
Overview of common approaches to electrochemical-regulation of depolymerization and polymer degradation.

**Scheme 3:**
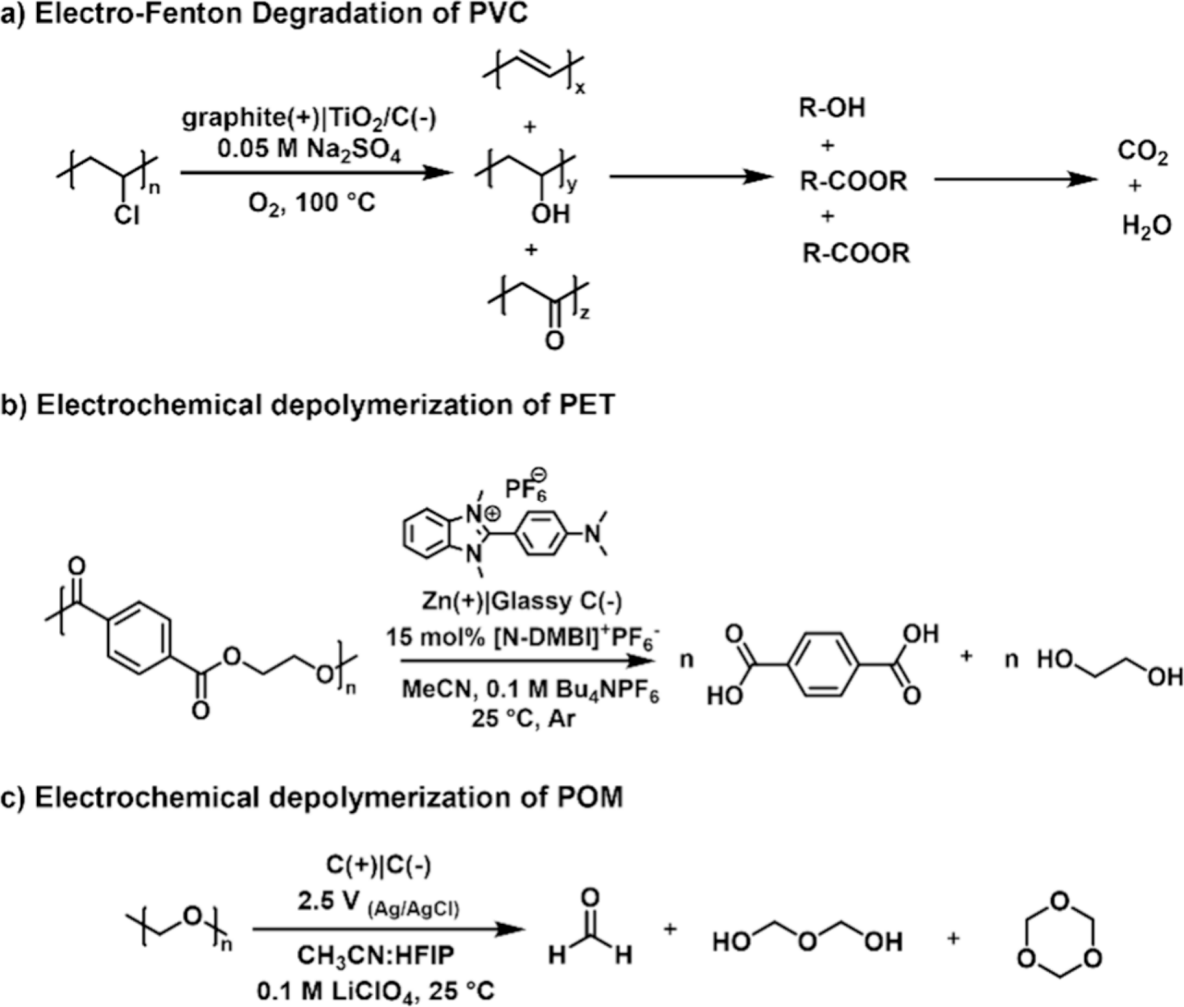
(a) Electro-Fenton Degradation of PVC To Give Carbon Dioxide and Water; (b) Electrochemical Depolymerization of PET under Ambient Conditions; (c) Electrochemical Depolymerization of POM, HFIP (Hexafluoroisopropanol)

First and foremost, electrochemical depolymerization/degradation of polymers (Figure 2a) comprised of low polarity C–C bonds (e.g., vinyl polymers) is challenging, and direct depolymerization of such polymers back to monomers has not yet been reported by solely electrochemical means. Initial work has instead demonstrated degradation, often accompanied by oxidation, with the aim of selectively forming useful small molecules (Figure 2b). In 2022, Leshkov and co-workers used an electrochemically mediated HAT method to activate inert C–C bonds at ambient conditions to degrade PS.^55^ In this study, *N*-hydroxy phthalimide (NHPI) was used as a mediator in a HAT reaction with benzylic C–H bonds, resulting in carbon-centered radicals which react with molecular oxygen to yield oxidative degradation of the PS resulting in several oxygenated products containing one or more phenyl rings. While the yield was relatively low (12%) in this proof-of-concept study, the authors noted significant potential for improved mass transfer through mixed solvents and vigorous stirring. Gao and co-workers developed an electro-Fenton like method using a TiO_2_/graphite cathode to degrade poly(vinyl chloride) microplastics.^56^ The process involved dechlorination through applied cathode potential and chain cleavage via hydroxyl radical oxidation, breaking PVC down into small organic molecules such as CO_2_ and H_2_O (Scheme 3a). The study achieved 56 wt % removal of PVC microplastics and 75% dechlorination efficiency after 6 h of electrolysis at −0.77 V vs Ag/AgCl at 100 °C. Degradation of microplastics is an intriguing example of a potential application of electrochemical degradation, and the technique has potential for other polymers such as polyethylene, polypropylene (PP), and PS. However, degradation with hydroxyl radicals does not typically generate particularly useful products, raising the question of whether further electrochemical modification of the dechlorinated polymers to give functionalized polymeric materials could be another viable alternative.

Another approach to degradation is to utilize electrochemistry to activate specifically incorporated functional groups to promote chain scission (Figure 2c). Recently, Sumerlin and co-workers demonstrated an approach for postpolymerization modification and degradation of polyacrylates using redox-active phthalimide groups.^57^ By integrating *N*-methacryloxyphthalimides units (PhthA) units into copolymers with MMA, they achieved quantitative olefin-methacrylate production under electrochemical conditions (2.5 V, C electrodes, 24 h) with an H atom donor (dithiothreitol). In the absence of the H atom donor, the PhthA-*co*-PMMA copolymer displayed significant degradation via both β-scission and decarboxylation, showing 95% degradation efficiency with just 1% PhthA. The degradation efficiency of this method surpasses other photoredox degradation strategies yet is limited to polymers that can incorporate redox-active units.

**Scheme 4:**
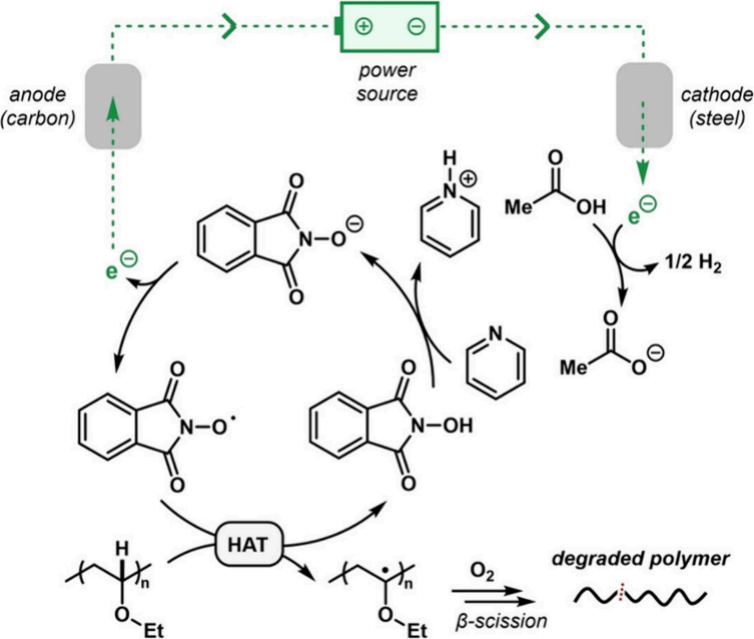
Proposed Catalytic Cycle of the NHPI-Mediated Degradation of Poly(ethyl vinyl ether) (Figure Partially Reproduced with Permission from Ref (63). Copyright 2023 Wiley-VCH)

In contrast to C–C bonds, electrochemical cleavage of C–O bonds has been extensively utilized for depolymerization/degradation, particularly on natural polymers such as lignin.^58,59^ Recently, this approach has also found use in depolymerization of synthetic polymers such as polyethylene terephthalate (PET). For example, Luca and co-workers used a H_2_O/MeOH electrolyte to generate a basic local environment to obtain as much as 17% terephthalic acid (TPA) from PET at room temperature.^60^ Additionally, Luca and co-workers also utilized benzimidazolium cations ([N-DMBI]^+^PF_6_^–^) as a redox mediator with a working potential of −2.25 V, to successfully obtain TPA and ethylene glycol (EG) repeating units from PET (Scheme 3b) with yields up to 90% yield and a faradaic efficiency greater than 90%.^61^ Depolymerization has also been demonstrated for polyethers, with Moore and co-workers reporting a mild electrochemical approach for depolymerizing highly crystalline polyoxymethylene (POM)^62^ using hexafluoroisopropanol (HFIP) as both a solvent and proton donor. This process enabled complete depolymerization at room temperature within 2 h, achieving 80% recovery of products including formaldehyde, oxidimethylene and 1,3,5-trioxane (Scheme 2c). Alternatively, Fors and co-workers introduced a cost-effective and mild strategy for degrading ether-containing polymers such as poly(propylene oxide) (PPO), poly(vinyl ether)s (PVEs), and polyurethanes (PU)^63^ via electrochemical C–H bond activation. This approach used NHPI as an electrochemical mediator, a C anode and steel cathode to selectively target polymers containing electron-rich C–H bonds (Scheme 4). Under optimized conditions (0.2 equiv of NHPI), PPO was degraded into acetic acid (14 mol %), formic acid (12 mol %), and acetaldehyde (4 mol %) after 25 h of electrolysis. Impressively they also demonstrated temporal control over PEVE degradation by switching the current on and off. However, polymers with electron deficient C–H bonds such as PMMA, PMA, and PVAc, remain intact.

In summary, electrochemically regulated degradation and depolymerization is an emerging field that has already shown promise in generating monomers and useful small molecules from common polymers under mild conditions, although has not yet been effective in directly regenerating monomer from vinyl polymers. We envision that much more of the electrochemistry developed for small molecules, particularly reactions which promote cleavage of C–C bonds,^64^ could be adapted and made suitable for polymeric substrates, which would yield significant advances. Electrochemical activation of polymer chain ends, analogous to photoactivation already reported for RDRP polymers, section 2, is also a potential avenue of research to gate the initiation of thermal depolymerization. Finally, the incorporation of redox-active units directly into polymers to facilitate degradation, as reported by Sumerlin, raises the question of whether other pristine polymers could be electrochemically modified to enhance degradability.

## 4 Mechanochemical Regulation

Mechanical force has long been recognized for its ability to degrade polymers through chain scission,^65,66^ a challenge that limits the efficacy of conventional mechanical recycling due to the resulting loss of material properties.^67,68^ However, recent advancements have sought to harness mechanochemical reactions for the targeted and controlled degradation and depolymerization of polymers.^69,70^ This section will focus on three key areas of research: polymer mechanochemistry, which involves incorporating mechanochemically labile elements into polymers; trituration mechanochemistry, where bond cleavage is typically induced through grinding in bulk materials, and sonochemistry, which uses ultrasound to break chemical bonds in solution.

### 4.1 Polymer Mechanochemistry

A common approach for achieving targeted degradation of polymers is through the incorporation of mechanophores; moieties which undergo specific chemical transformations under mechanical force, into the polymer backbone.^71^ These mechanophores can be used to create strategic cleavage sites, facilitating break down of the polymer into oligomeric fragments upon mechanical agitation.^72^ This concept is also sometimes referred to as gated degradability since prior activation is crucial.^73^

**Figure 3:**
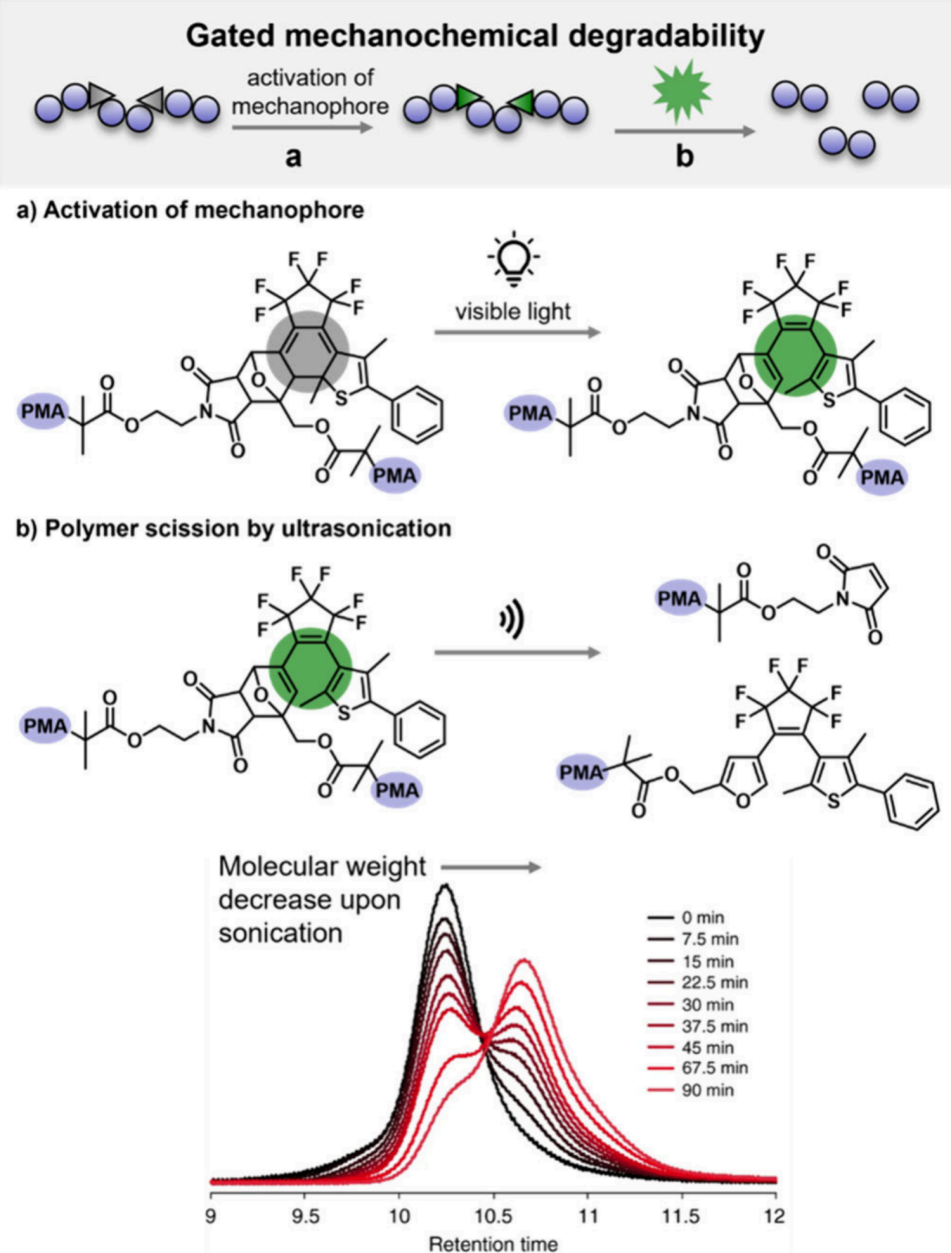
Gated mechanochemical degradation of PMA incorporating a mechanophore. (a) Activation of the mechanophore by visible light. (b) Scission of the mechanophore to break the polymer chain, yielding a decrease in molecular weight as observed by SEC.

Craig and co-workers developed a mechanophore system that could convert a single mechanochemical chain scission event into many by mechanochemical remodeling of the backbone. Consequently, they were able to degrade the polymer much below the molecular weight limit imposed by strain-initiated chain-scission.^74^ Instead of directly degrading the polymer through the mechanophore, Bruns and co-workers copolymerized vinyl monomers with modified cyclobutene units via radical polymerization.^75^ These cyclobutene units ring-opened to form ester or imide backbone bonds when the polymer was subjected to ultrasonication. The heteroatom bonds could then be cleaved by hydrolysis in a subsequent treatment under basic conditions. Similarly, Tang and co-workers demonstrated the mechanochemical activation of cyclobutene-fused succinimide (CBS) units in vinyl polymers, leading to degradation upon hydrolysis.^76^ Interestingly, this method can be applied to typically hard-to-process polymers like polyacrylates and polyacrylamides, which have high ceiling temperatures (*T*_c_s). In both of these examples, mechano-activation makes the polymer degradable by another chemical trigger, in this case a base. In such systems, the polymer potentially retains good stability even though it can be mechanically activated since both triggers have to be applied to degrade the polymers.^77^ The concept of gated mechanophores can also be applied in the reverse order, where the mechanophore has to be activated by another trigger first. Otsuka and co-workers developed a diarylethene-conjugated Diels–Alder adduct as a photoswitch, where the adduct becomes a mechanophore only once the polymer is subjected to UV light (Figure 3).^78^ This reactivity can subsequently be switched back by exposing the polymer to visible light. Thereby, mechanochemical degradation can be switched on by demand.

Although polymer mechanochemistry is a powerful technique for increasing the degradability of polymeric materials which could find use in specialized applications, there are a number of possible drawbacks in applying this to commercial polymers. Incorporation of mechanophores would increase costs and could potentially alter the physical properties of the polymer, affecting parameters such as tensile strength and glass transition temperatures. Additionally, degradation of polymers into oligomers still poses the problem of how to reuse/recycle the material.

### 4.2 Trituration Mechanochemistry

**Figure 4:**
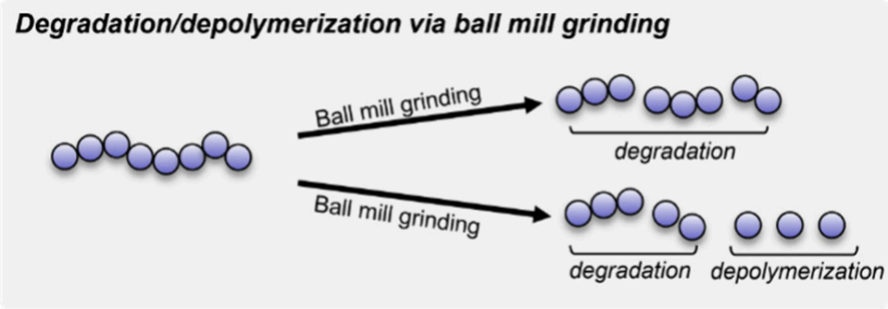
Ball mill grinding of polymers can yield both degradation via chain scission and depolymerization.

Trituration mechanochemistry is defined as the promotion of chemical reactions by the absorption of mechanical energy, usually through a grinding or milling process.^73^ When applied to polymeric materials, this often results in chain scission and the formation of oligomers, similar to the mechanophore examples in the previous section, albeit in a less directed manner (Figure 4). The extent of chain-scission under milling conditions is highly dependent on the molecular weight of materials, as well as the *T*_g_.^79^ For polyesters, which contain heteroatoms along the polymer backbone, trituration mechanochemistry has been employed to achieve efficient depolymerization. For example, mechanochemical milling of PET with NaOH has been shown to yield near quantitative monomer conversions under solvent-free, ambient conditions.^80,81^

As for vinyl polymers, which are comprised of C–C backbones, Kim, Peterson, Choi, and co-workers demonstrated that PMS undergoes significant depolymerization after just a few minutes of ball-mill grinding, yielding 64% monomer.^82^ Simulations and radical trapping experiments suggest that depolymerization is initiated by chain scission, generating radicals capable of depropagation. Notably, α-methyl styrene has a relatively low bulk *T*_c_, making it susceptible to depolymerization under mild conditions. However, higher *T*_c_ polymers like PMMA and PS were also shown to depolymerize under similar grinding conditions, though with much lower yields of 4% and 1% monomer, respectively. More recently, Peterson and Choi reported that polymethacrylates such as PMMA could achieve >40% depolymerization when milled for 8 min at just 43 °C using steel balls in the presence of oxygen. These results are significant because the milling experiments were conducted close to ambient temperature, challenging previous assumptions about the thermal requirements for depolymerization.^23,83^

**Scheme 5:**
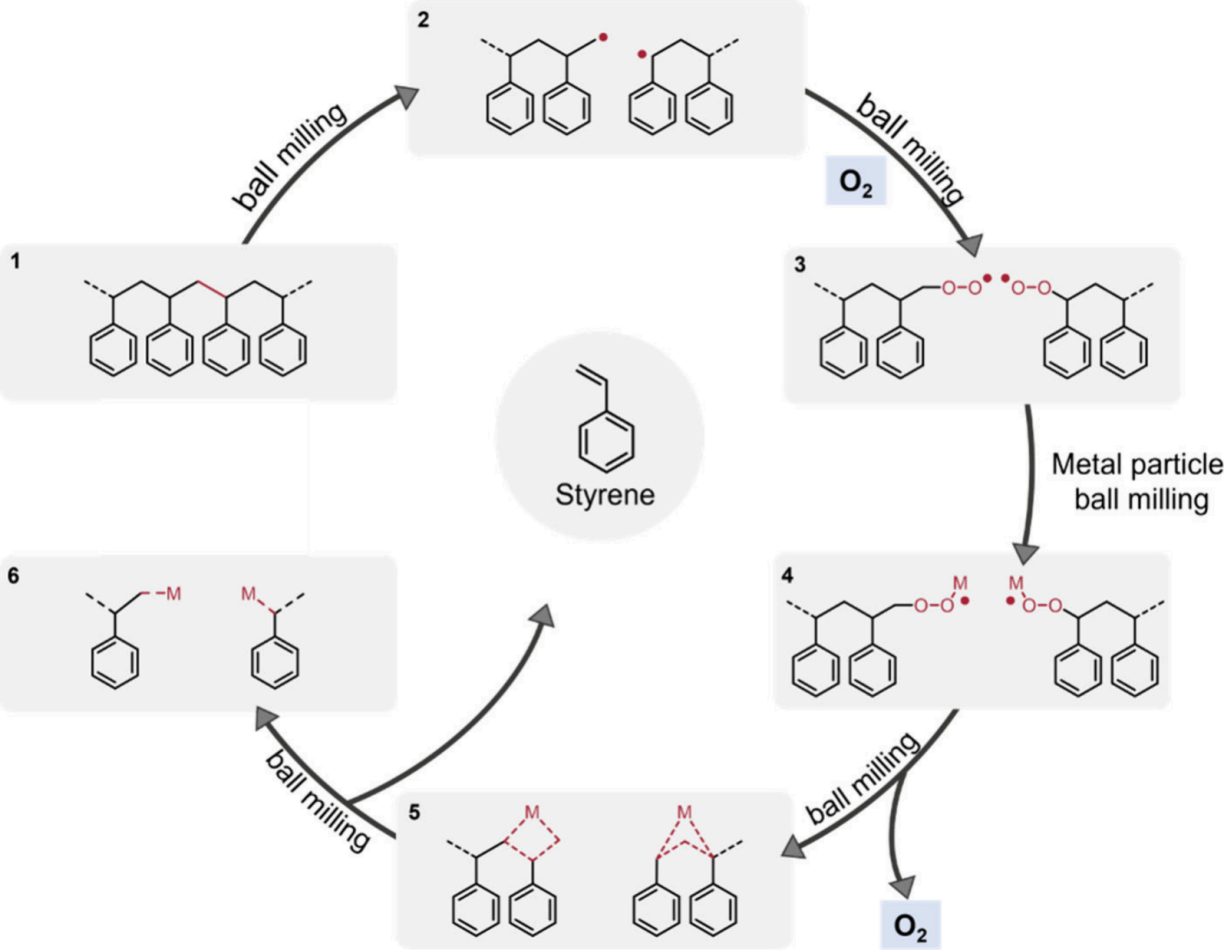
Proposed Mechanism of Monomer Generation in the Ball Milling of PS in the Presence of Metallic Milling Media and Oxygen

Similarly, Balema, Luzinov, and co-workers reported a notable case of PS depolymerization under ambient conditions.^84^ Using ball-mill grinding with either stainless steel or tungsten carbide balls in the presence of air, they recovered approximately 7 wt % of monomeric styrene. Although the yield was modest, it was unexpectedly higher than anticipated given that the experiments were conducted at room temperature. To exclude the possibility of thermal effects, the researchers performed a control experiment by milling a mixture of PS and ammonium carbonate, which decomposes at around 60 °C. The lack of thermal decomposition products confirmed that the depolymerization was mechanochemical in nature. The proposed mechanism (Scheme 5) involves the scission of polymer chains, forming carbon-centered radicals. These radicals react with atmospheric oxygen to form peroxide intermediates, which subsequently interact with the metallic surfaces of the milling media, catalyzing further depolymerization and monomer release. A further study by Sievers and co-workers also indicated a catalytic role of both iron and oxygen in promoting monomer production during ball mill grinding of PS and confirmed that styrene is the major product.^85^

Catalytic ball milling has recently been employed for the depolymerization of PP, a polyolefin that poses significant challenges for recycling due to its high chemical stability.^86^ Pyrolysis of PP yields a complex mixture of hundreds of different hydrocarbons because of random chain-scission at elevated temperatures. Vollmer and co-workers utilized ball milling with surface-activated zirconia balls, referred to as “surface-activated mechano-catalysts” (SAM catalysts) to achieve depolymerization and degradation under ambient conditions. In contrast to catalytic cracking of PP, which proceeds via a cationic mechanism, the catalytic ball milling process is radical in nature. Mechanical energy initiates bond scission in the PP chains, followed by controlled radical reactions that yield a more focused range of small hydrocarbons (C_1_–C_10_), including propene, methane, and ethane. Compared to untreated zirconia balls, the SAM catalysts dramatically increase the hydrocarbon yield, achieving 45% conversion within 1 h of milling at room temperature. This study further highlights that while radical mechanisms can typically cause random degradation, the use of catalysis in ball milling offers a novel, low-energy pathway to control these reactions and convert plastic waste into valuable products.

Trituration mechanochemistry is evolving into a compelling area of study with significant implications for polymer recycling. Recent advancements indicate that mechanical energy can promote chain scission while also introducing catalytic^86^ and potentially even thermodynamic effects^85^ that enhance depolymerization, processes which are not yet fully understood and require further study. Notably, the successful depolymerization of PS under ambient conditions demonstrates that monomer regeneration can occur at low temperatures, challenging conventional assumptions about the thermal requirements for this process. Moreover, the use of catalytic milling media highlights a promising pathway to convert challenging plastic waste into valuable hydrocarbons at room temperature. Finally, it is worth noting that ball milling can be successfully employed on large scales,^87^ suggesting a practical route for integrating these mechanochemical strategies into recycling practices.

### 4.3 Sonochemistry

**Figure 5:**
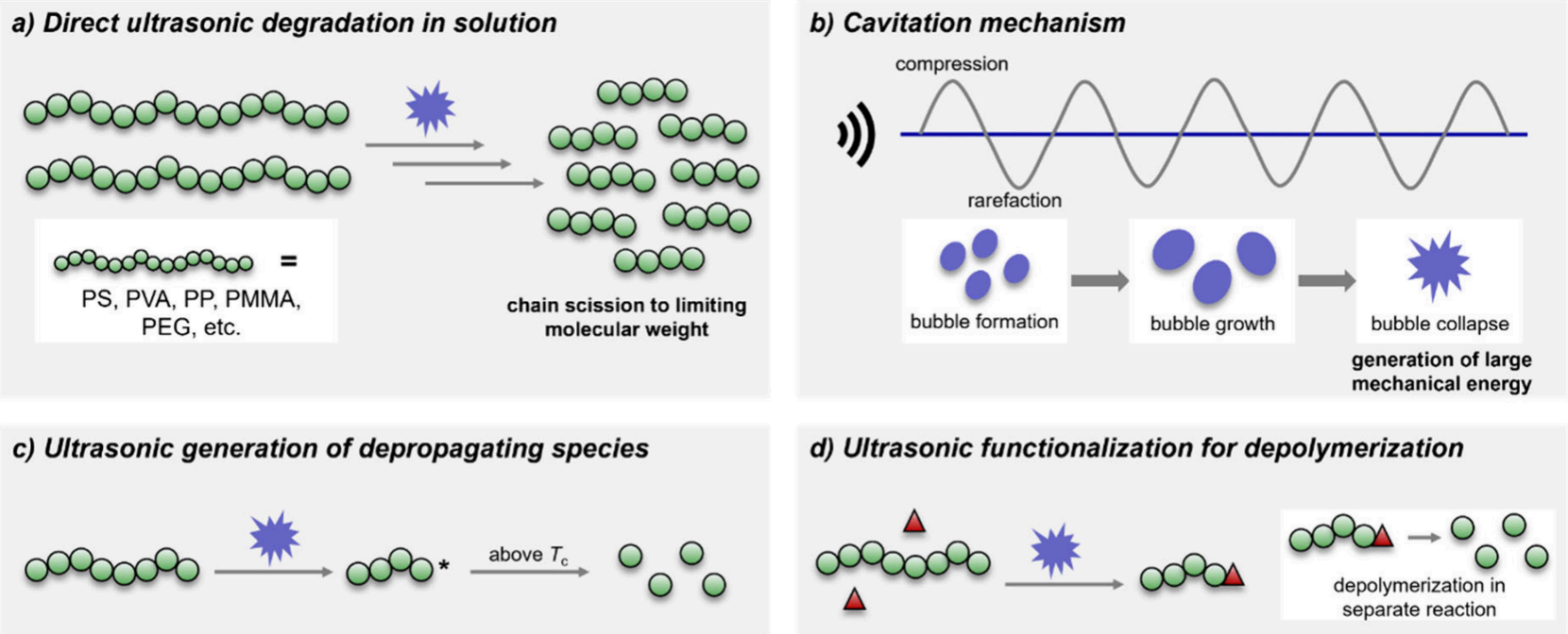
(a) Chain scission to limiting molecular weight from sonication. (b) Scheme depicting a simplified cavitation mechanism for the generation of mechanical energy under sonication. (c) Generation of active species capable of depropagating via sonication. (d) Ultrasonic functionalization of polymers to enhance depolymerizability.

Ultrasonic degradation of polymers in solution has been a known phenomenon since the beginning of polymer research, when researchers already observed that the viscosity of polymer solutions dropped when they were subjected to ultrasonic radiation.^88^ Since then, direct polymer degradation by the application of nondirected, high-energy sonication (Figure 5a) has been reported for many different polymer classes and types such as polyolefins, vinyl polymers, polyesters, polyethers, PUs, and biopolymers such as lignin, and starch.^89−93^

Degradation of polymers via ultrasonication in solution occurs via mechanically induced scission around the middle of the polymer chain. The energy input from the sonication alone is not high enough to result in direct breakage of any backbone bonds; polymers do not degrade when directly exposed to ultrasound in bulk. Rather, bond breakage occurs due to strain on the chain as a result from sonication-induced cavitation: the acoustic waves result in the formation of microbubbles in the solution when impurities such as dissolved gases are present. These bubbles undergo oscillatory expansion/contraction driven by acoustic pressure,^94^ and finally collapse, releasing a high amount of mechanical energy into the solution (Figure 5b). Degradation occurs as a result of acceleration gradient from bubble collapse rather than hot-spots,^95^ and therefore depends heavily on factors such as the solvents’ physical properties and the type and amount of dissolved gas dissolved,^96^ the pressure in the reaction vessel, the physical properties of the polymer,^97^ and the solution temperature.^98^ Generally, the higher the energy input, the more a polymer will degrade and the smaller the remaining fragments. The remaining fragments are the main limiting factor in direct ultrasonic degradation. Polymer chains generally tend to break toward the middle since the strain is the highest there. During every degradation reaction, a point is reached, at which no more breakage occurs as the introduced strain is not sufficient to break backbone bonds. The nature of the reaction therefore always results in the presence of oligomeric species and breaking the polymer down into small molecules or monomer is generally not possible.

**Scheme 6:**
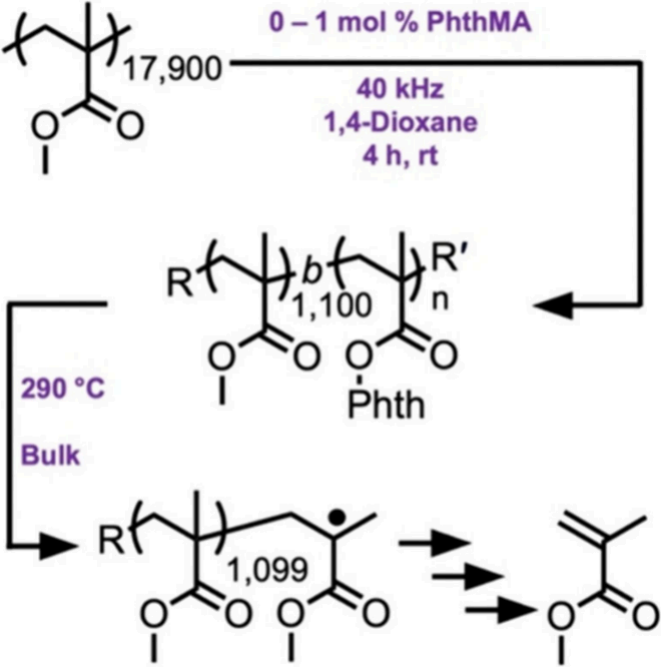
Reaction Scheme for the Functionalization of PMMA by Sonication in the Presence of PhthMA (Figure Partially Reproduced with Permission from Ref (100). Copyright 2024 Wiley-VCH)

One option to regenerate monomer through the application of ultrasound is to generate an active species through sonication, which then depropagates when the polymer is kept above its chemical equilibrium conditions (concentration below the monomer equilibrium concentration and temperature above the *T*_c_, Figure 5c).^23^ Such work was demonstrated by Moore and co-workers, who depolymerized poly(*o*-phthalaldehyde), a low *T*_c_ polymer, in solution after generating the depropagating ionic species through heterolytic chain scission induced by ultrasonication.^99^ While the approach is straightforward, most polymers require significantly enhanced temperatures to depropagate in solution, which strongly reduces the cavitation efficiency. To overcome this problem, Sumerlin and co-workers used ultrasound to install a thermally degradable phthalimide unit onto commercial PMMA (Figure 5d). By sonicating the polymer in the presence of a phthalimide-functionalized monomer, the monomer was added onto the chain-scissioned polymer fragment. The resulting polymer could subsequently be thermally depolymerized in bulk at elevated temperatures (Scheme 6).^100^ Such a strategy has also been employed in the past to functionalize methacrylate polymers with various end-groups,^101,102^ which could potentially also be activated for depolymerization by other reported RDRP depolymerization methodologies in a similar manner.

Sonication has also been utilized to increase the rate of depolymerization or degradation reactions that would overwise be slow. For polyesters, including polycaprolactone (PCL), ester bond hydrolysis could be significantly improved when the backbone bonds were agitated with sonication. Similar treatment of polymers with orthogonal ester bonds in the side chains e.g. PMMA were not affected since they were not susceptible to the same mechanical forces.^103^

Recent advancements in ultrasonication for depolymerization reveal that sonication can offer a more controlled method for polymer degradation compared to the conventional approach of relying solely on cavitation-induced chain scission. By moving beyond the traditional focus on midchain scission resulting from mechanical strain, this technique allows for the effective breakdown of polymers into smaller molecular products, and in some instances, even to their constituent monomers. While many of these innovative methodologies utilize affordable and accessible benchtop sonicator baths, the effectiveness of these techniques often hinges on the use of specialized materials and specific chemical moieties. Consequently, despite the promise of these methods, the existing literature on their application remains limited. This gap highlights a significant opportunity for future research to explore and exploit these principles more thoroughly, potentially leading to enhanced and more sustainable polymer recycling methods.

## Outlook

As the global demand for polymeric materials continues to rise, innovations in polymer degradation and depolymerization via external regulation represent promising potential in combatting the environmental problems posed by polymer waste. Current advancements in the use of stimuli such as light, electrochemistry, and mechanical force have already begun to demonstrate selectivity in breaking polymers down into useful small molecules or even regenerating monomers under mild conditions. However, substantial challenges remain to translate these initial laboratory successes into tangible change in polymer sustainability. Critically, for any of these approaches to be truly viable for addressing plastic waste, their lifecycle analysis (LCA) must demonstrate a favorable environmental and economic outcome compared to conventional pyrolysis methods. While a comprehensive discussion of LCA is beyond the scope of this review, it is likely to become a critical factor in the field as more efficient processes are developed. Without a positive LCA, even the most elegant and efficient externally regulated strategies may struggle to offer a viable alternative to current plastic recycling technologies. To achieve meaningful progress toward sustainability, polymer chemists would greatly benefit from close collaboration with LCA experts. Such partnerships could help identify the most beneficial target products from specific plastic wastes, ensuring that depolymerization strategies are not only technically elegant but also aligned with broader sustainability goals. For example, generation of small molecules/oligomers from polymers by degradation is only beneficial if there is a need for such products. By integrating lifecycle analysis into the development of externally regulated degradation processes, the field can move beyond theoretical advances toward impactful, real-world solutions for the plastic waste crisis.

Scaling up these reactions is a critical step for transitioning from laboratory successes to commercial viability. The use of solvents, while often necessary in polymer degradation, can present challenges for green processes due to environmental and economic concerns. Mechanochemical methods, which are typically conducted without solvents, offer a clear advantage in this regard. For processes where solvents cannot be avoided, flow chemistry provides a promising alternative by improving efficiency, enabling continuous operation, and facilitating solvent recycling. These strategies will be essential in overcoming scale-related barriers and ensuring that externally regulated polymer degradation technologies are both practical and sustainable on an industrial scale.

Photoregulation has demonstrated significant promise in polymer degradation, particularly through the use of photothermal methods and photocatalysis. However, the development of more selective light-promoted HAT methods which target protons on specific backbone carbons would allow for controlled and targeted degradation, significantly improving the purity of the recovered monomers or small molecules. Transitioning photoinduced reactions from batch to continuous flow processes will be essential for real-world applications but could also allow for higher efficiency by improving light penetration and enabling more uniform photochemical reactions. Exploring the integration of these systems with renewable energy sources, such as solar-driven photocatalysis, could further enhance the sustainability of these processes.

Electrochemical regulation is still an emerging field in polymer degradation/depolymerization, and further rapid growth is expected. Although significant strides have been made with polymers with C–O bonds, much of the focus on C–C backbone polymers has been on oxidation or modification rather than full depolymerization. To advance this field, the application and adaption of electrochemical methods capable of breaking strong C–C bonds, which are prevalent in many commercial polymers like polystyrene and polyolefins, must be realized.

Mechanochemical regulation offers some of the most intriguing potential due to its energy efficiency and the ability to perform reactions without the need for solvents or elevated temperatures. Early studies in depolymerization of vinyl polymers have shown promising results, especially with catalytic ball milling. However, the mechanistic implications of these processes are not yet fully understood, and further exploration is necessary. Detailed mechanistic studies could uncover new ways to harness mechanical force more effectively and understand the catalytic interactions that appear to be taking place under certain conditions.

Although chemical degradation strategies have not been addressed in detail in this perspective, recent work toward this direction holds particular promise and may be further explored in the next years.^104−108^

In conclusion, while photo, electrochemical, and mechanochemical regulation each offer distinct advantages, they all require advancements in selectivity, scalability, and mechanistic insight. Tackling these challenges will be critical for future research to unlock their full potential, enabling more efficient, cost-effective, and sustainable polymer recycling methods that drive the transition toward a circular economy.
